# Phytochemical Characterization and Immunometabolic Modulation by *Mangifera indica* (Mahajanaka) Pulp Extract in Diabetic and Hypertensive Rat Models

**DOI:** 10.3390/ijms27135742

**Published:** 2026-06-25

**Authors:** Wachiraporn Tipsuwan, Hathairat Thananchai, Anusara Pongjanta, Suphatta Yubo, Tawat Taesothikul, Duangta Kanjanapothi, Yanping Zhong, Somdet Srichairatanakool

**Affiliations:** 1 Division of Biochemistry, Faculty of Medical Sciences, University of Phayao, Phayao 56000, Thailand; wachiraporn.ti@up.ac.th; 2 Department of Microbiology, Faculty of Medicine, Chiang Mai University, Chiang Mai 50200, Thailand; hathairat.t@cmu.ac.th; 3 Department of Biochemistry, Faculty of Medicine, Chiang Mai University, Chiang Mai 50200, Thailand; em_anusara_p@crru.ac.th (A.P.); supattar0210@gmail.com (S.Y.); yanping_z@cmu.ac.th (Y.Z.); 4 School of Health Science, Chiang Rai Rajabhat University, Chiang Rai 57100, Thailand; 5 Faculty of Pharmacy, Payap University, Chiang Mai 50000, Thailand; tawattae@gmail.com (T.T.); duangtakan@gmail.com (D.K.); 6 School of Medical Technology and Artificial Intelligence, Youjiang Medical University for Nationalities, Baise 533000, China

**Keywords:** mango, *Mangifera indica*, diabetes, blood pressure, phagocytosis, cytokines, immunomodulatory

## Abstract

Mango (*Mangifera indica* L.) pulp contains bioactive compounds with potential therapeutic effects against metabolic and immune-related disorders. However, the integrated effects of mango pulp extract on metabolic, cardiovascular, and immunomodulatory functions remain insufficiently characterized. Therefore, this study aimed to characterize the phytochemical composition and evaluate the activity of *M. indica* (Mahajanaka) pulp ethanolic extract (MPEE) in rat models. Streptozotocin (STZ)-induced diabetic rats and N(G)-nitro-L-arginine methyl ester hydrochloride (L-NAME)-induced hypertensive rats were used to assess metabolic and cardiovascular effects, while immune function was examined through neutrophil phagocytosis, splenocyte proliferation, lymphocyte subpopulation analysis, and cytokine secretion. MPEE exhibited a rich phytochemical profile, particularly phenolic compounds, along with strong antioxidant activity (339 ± 8.9 mg gallic acid equivalent/g extract). In STZ-induced diabetic rats, MPEE at 300 mg/kg significantly reduced plasma triglyceride (36.7%) and total cholesterol (45.3%) levels compared with untreated diabetic controls, although its antihyperglycemic effect was modest (6.8%). In L-NAME-induced hypertensive rats, MPEE at 400 mg/kg produced the greatest reduction in blood pressure (42.7%) and heart rate (53.5%). Furthermore, MPEE enhanced neutrophil phagocytic activity (43%), with significant increases observed at doses of 100–400 mg/kg. These findings indicate that MPEE exerts antioxidant, hypolipidemic, antihypertensive, and innate immunostimulatory activities.

## 1. Introduction

Chronic metabolic disorders, including diabetes mellitus, dyslipidemia, and hypertension, represent major global health challenges and are closely associated with increased morbidity and mortality rates [1,2,3,4]. Current pharmacological management of diabetes relies on agents such as metformin, sulfonylureas, sodium-glucose cotransporter-2 inhibitors, glucagon-like peptide-1 receptor agonists, and insulin [5]. In addition, hypertension is commonly treated using angiotensin-converting enzyme inhibitors, angiotensin receptor blockers, calcium channel blockers, β-blockers, and diuretics [6,7]. Although these therapies are generally effective, their long-term use may be associated with adverse effects and reduced treatment adherence in some patients [8]. Consequently, there is growing interest in identifying natural products with multi-target biological activities that may serve as complementary approaches for the prevention and management of chronic metabolic and cardiovascular disorders. These conditions are often interconnected and have also been linked to oxidative stress, inflammation, and immune dysregulation, which together contribute to disease progression and other related complications. In recent years, there has been growing interest in identifying natural products with multi-target therapeutic potential, particularly those capable of modulating both metabolic and immune functions [1].

*Mangifera indica* L. (mango) is a widely consumed tropical fruit known not only for its nutritional value but also for its pharmacological properties. Various parts of the mango plant, including the leaves, bark, and fruit pulp, have been reported to possess antioxidant, anti-inflammatory, antidiabetic, and cardioprotective activities [2]. Among its bioactive constituents, mangiferin (MGF), a xanthonoid polyphenol, has been extensively studied for its beneficial effects against lifestyle-related disorders, including diabetes, cardiovascular diseases, and metabolic syndrome [1]. Previous studies have demonstrated that MGF and mango extracts can improve glucose metabolism, enhance insulin sensitivity, and reduce lipid accumulation through the modulation of oxidative stress and inflammatory pathways [1,3,9,10,11]. In addition to its metabolic effects, mango-derived compounds have also been reported to exert certain cardiovascular protective effects, including antihypertensive activity. Experimental studies have shown that plant-derived polyphenols can improve vascular function by enhancing nitric oxide bioavailability and reducing oxidative damage [12,13]. These mechanisms are particularly relevant under certain conditions, such as hypertension, where endothelial dysfunction plays a critical role. Despite these findings, the combined evaluation of metabolic and cardiovascular effects of mango pulp extracts remains limited.

Interestingly, natural products can influence the immune system, enhance macrophage and neutrophil activity, and regulate lymphocyte proliferation and cytokine production [4,10,14,15,16]. The ability to modulate immune responses is particularly important in chronic diseases, where immune imbalance contributes to disease progression. Moreover, cytokines such as interferon gamma (IFN-γ) and interleukin 4 (IL-4), serve as key indicators of T-helper cells 1 and 2 (Th1/Th2) balance, which plays a central role in maintaining immune homeostasis [17,18]. However, the immunomodulatory effects of mango pulp extract, particularly in the context of long-term administration, remain insufficiently characterized [19]. Although previous studies have reported the antioxidant and biological activities of Mahajanaka mango pulp extract in murine models [20], there is a lack of comprehensive studies that have evaluated its combined effects on metabolic, cardiovascular, and immune functions. In particular, the relationship between these systems and the potential of mango pulp extract to act as an immune–metabolic modulator has not been fully explored.

Although numerous studies have reported the antidiabetic, antioxidant, anti-inflammatory, and cardiovascular activities of *M. indica* extracts and MGF, most have focused on individual biological effects. To the best of our knowledge, few studies have comprehensively examined the relationships among metabolic regulation, cardiovascular protection, and immune modulation using Mahajanaka mango pulp extract. Moreover, information regarding its effects on neutrophil phagocytic function, lymphocyte subpopulations, splenocyte proliferation, and cytokine production remains limited. Therefore, the present study aimed to investigate the phytochemical composition and immunometabolic modulatory effects of MPEE in rats. Specifically, this study evaluated metabolic parameters, cardiovascular responses, neutrophil phagocytic activity, splenocyte subpopulations, lymphocyte proliferation, and cytokine secretion. By integrating these outcomes, this study provides a novel and comprehensive assessment of the biological activities of MPEE and its potential as a multi-target natural therapeutic agent.

## 2. Results

### 2.1. Chemical Compositions and Contents, and Antioxidant Activity in MPEE

#### 2.1.1. Phenolic Compounds and Their Derivatives

**Figure 1:**
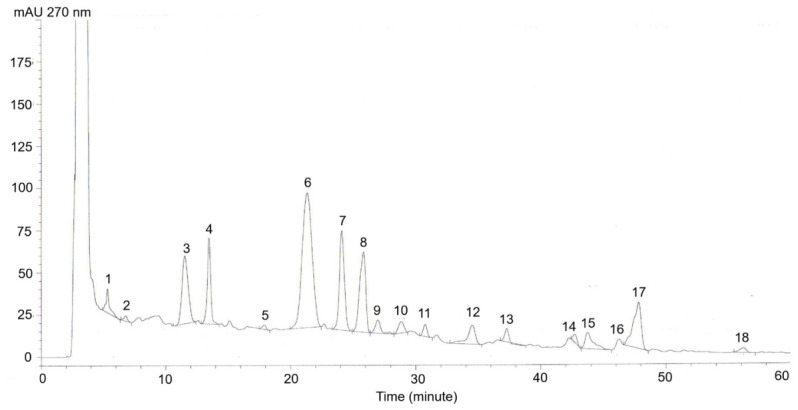
Representative high-performance liquid chromatography (HPLC) (UV detection at 270 nm) and corresponding electrospray ionization–mass spectrometry (ESI–MS) spectra of *Mangifera indica* (Mahajanaka) pulp ethanolic extract (MPEE) showing major peaks and their associated mass-to-charge ratio (*m*/*z*) values obtained in positive ionization mode.

High-performance liquid chromatography/electrospray ionization–mass spectrometry (HPLC/ESI-MS) analysis of the MPEE revealed a complex chromatographic profile comprising 18 chromatographic peaks detected over a retention time (T_R_) range of approximately 5 to 56 min (Figure 1 and Table 1).

Based on T_R_ and mass-to-charge (*m*/*z*) values, these peaks were tentatively assigned to MGF-related xanthones, flavonoids (catechin/epicatechin-like compounds and quercetin glycosides), benzophenone glycosides, phenolic acids, and other polyphenolic derivatives. Among these, MGF-related xanthones and flavonoid glycosides represented the predominant phytochemical classes detected in the extract.

#### 2.1.2. Phenolic, Mangiferin, Ascorbic Acid, β-Carotene Contents, and Antioxidant Activity

According to our chemical composition analysis (Table 2), the MPEE exhibited levels of total phenolic content (TPC) (339 ± 8.9 mg GAE/g), mangiferin (0.55 ± 0.96 mg/g), L-ascorbic acid (0.39 ± 0.13 mg/g) and β-carotene (5.87 ± 0.06 mg/g). In addition, the MPEE demonstrated scavenging activity against 2,2-azino-di-[3-ethylbenzthiazolinsulfonate (ABTS) radicals, with 740 ± 8 mg Trolox-equivalent (TE)/g.

### 2.2. Antidiabetic and Hypolipidemic Effects in STZ-Induced Rats

The experimental results in Table 3, Table 4 and Table 5 show levels of fasting blood glucose (FBG), plasma triglyceride (TG) and total cholesterol (TC), respectively, in normal (N) rats and diabetic mellitus (DM) rats induced by intraperitoneal (ip) injection of STZ at 3 mg/kg body weight (BW). As is shown in Table 3, FBG levels remained stable in N rats throughout the course of the study, whereas DM rats (control) exhibited persistently elevated FBG concentrations. One-way analysis of variance (ANOVA) revealed a highly significant difference in FBG levels among experimental groups at week 6 (F = 131.08, *p* < 0.001). Post hoc analysis using Tukey’s honestly significant difference (HSD) test demonstrated that glibenclamide (GBA) treatment (3 mg/kg BW/day) significantly reduced FBG levels when compared with DM rat controls that had been treated with deionized water (DI) (*p* < 0.001). Similarly, yellow MPEE at 300 mg/kg produced a significant reduction (*p* < 0.01), whereas the 30 mg/kg dose did not result in a statistically significant change. These findings confirm the dose-dependent antihyperglycemic effect of MPEE. Within-group analysis revealed no significant changes in FBG levels in normal rats or diabetic control rats throughout the experimental period. In contrast, GBA treatment produced a significant reduction in glucose levels at weeks 4 and 6 (*p* < 0.05–0.01). MPEE treatment showed minimal effects, with only the higher dose demonstrating a modest reduction at week 6 (*p* < 0.05), while the lower dose did not produce any significant changes. These findings suggest that MPEE exhibits limited antihyperglycemic activity when compared with GBA.

Plasma TG levels were significantly elevated in diabetic control rats when compared with normal rats. Treatment with GBA resulted in a marked reduction in TG levels (*p* < 0.001). Similarly, MPEE treatment, particularly at the higher dose, significantly decreased TG concentrations when compared with diabetic controls (Table 4). These findings indicate that MPEE improved lipid metabolism in diabetic rats in a dose-dependent manner. Within-group analysis revealed no significant changes in TG levels in normal rats or diabetic control rats across the study period. In contrast, GBA treatment produced significant reductions at weeks 2, 4, and 6, indicating consistent improvement in lipid metabolism. MPEE treatment demonstrated significant reductions at early time points, particularly at weeks 2 and 4, with moderate effects sustained at later stages. These findings suggest that MPEE can exert time-dependent and dose-related antihyperlipidemic effects, although its efficacy is less consistent than GBA.

Plasma TC levels differed significantly among experimental groups at all time points, as shown by one-way ANOVA at week 0 (F = 5.21, *p* < 0.001), week 2 (F = 3.43, *p* < 0.01), week 4 (F = 3.96, *p* < 0.01), and week 6 (F = 4.03, *p* < 0.01). Tukey post hoc analysis using diabetic control rats as the reference group showed that significant between-group differences were most evident at week 6, where GBA and both MPEE-treated diabetic groups exhibited lower total cholesterol levels than untreated diabetic rats (Table 5). Within-group comparisons relative to the baseline further indicated that diabetic control rats did not experience a significant reduction in TC over time, whereas GBA produced a significant decrease in TC by week 6 (*p* < 0.01). High-dose MPEE also reduced TC significantly at week 6 (*p* < 0.05), indicating a delayed but beneficial hypocholesterolemic effect. Overall, these findings suggest that MPEE improved cholesterol homeostasis in diabetic rats, with the higher dose showing a stronger late-stage response, although the effect remained less consistent than that of GBA.

### 2.3. Effect of MPEE Treatment of Antioxidant Activity

Plasma total antioxidant capacity (TAC) exhibited modest variations across experimental groups over time. No significant changes were observed in normal or diabetic control rats. Treatment with GBA and MPEE resulted in slight increases in antioxidant activity at later time points, although these changes were less pronounced when compared with alterations observed in glucose and lipid parameters (Table 6). These findings suggest that while MPEE may exert antioxidant effects, its primary impact appears to be on glycemic and lipid regulation.

### 2.4. Effects of MPEE Treatment on Blood Pressure and Heart Rate of Normotensive Rats

**Figure 2:**
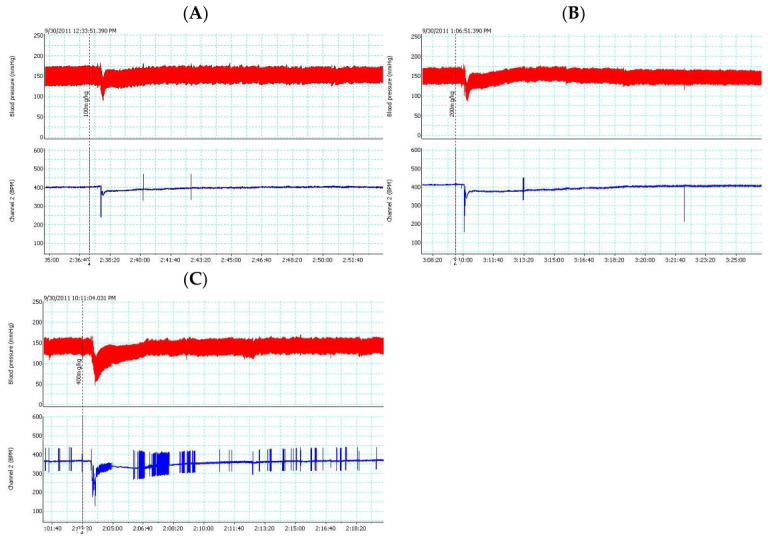
Effects of MPEE on blood pressure (BP) (red tracers) and heart rate (HR) (blue tracers) in normotensive rats. Rats were anesthetized with phenobarbital and treated MPEE at doses of 100 mg/kg (**A**), 200 mg/kg (**B**), or 400 mg/kg (**C**). Changes in BP and HR were monitored over time. Abbreviations: MPEE, *Mangifera indica* (Mahajanaka) pulp ethanolic extract.

Administration of MPEE (100, 200, and 400 mg/kg) produced dose-dependent changes in cardiovascular parameters in normotensive rats (Figure 2A, 2B and 2C, respectively) Blood pressure (BP) expressed in mm Hg showed a gradual reduction following treatment, particularly at higher doses, while heart rate (HR) expressed in beats per min (bpm) exhibited moderate fluctuations. These effects suggest that MPEE may exert mild cardiovascular activity, although the magnitude of change was limited under normotensive conditions.

### 2.5. Effects of MPEE Treatment on BP and HR in Hypertensive Rats

**Figure 3:**
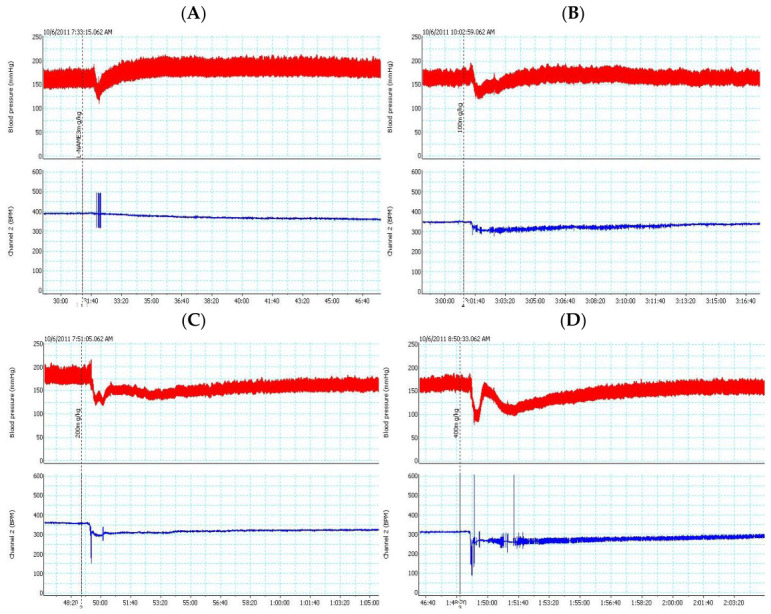
Effects of MPEE on blood pressure (red tracers) and heart rate (blue tracers) in N(G)-nitro-L-arginine methyl ester hydrochloride (L-NAME)-induced hypertensive rats. Hypertension was induced using intravenous injection of L-NAME (dot lines) (3 mg/kg), followed by treatment with DI (**A**) or MPEE at doses of 100 mg/kg (**B**), 200 mg/kg (**C**), and 400 mg/kg (**D**). Changes in blood pressure and heart rate were monitored over time. Abbreviations: bpm, beat per minute; DI, deionized water; MPEE, *Mangifera indica* (Mahajanaka) pulp ethanolic extract.

Administration of L-NAME produced a marked increase in BP, confirming successful induction of hypertension. Thus, as shown in Figure 3A–D, treatment with varied doses of MPEE resulted in a dose-dependent reduction in BP, with the highest dose (400 mg/kg) producing the most pronounced effect when compared with hypertensive controls. Moderate reductions were observed at 200 mg/kg, while the lowest dose exhibited minimal effects. HR showed less pronounced changes, with only modest alterations following treatment. These findings indicate that MPEE exerted significant antihypertensive activity, particularly at higher doses, with a stronger effect on BP than HR.

### 2.6. Effects of MPEE Treatment on Neutrophil Phagocytotic Activity

**Figure 4:**
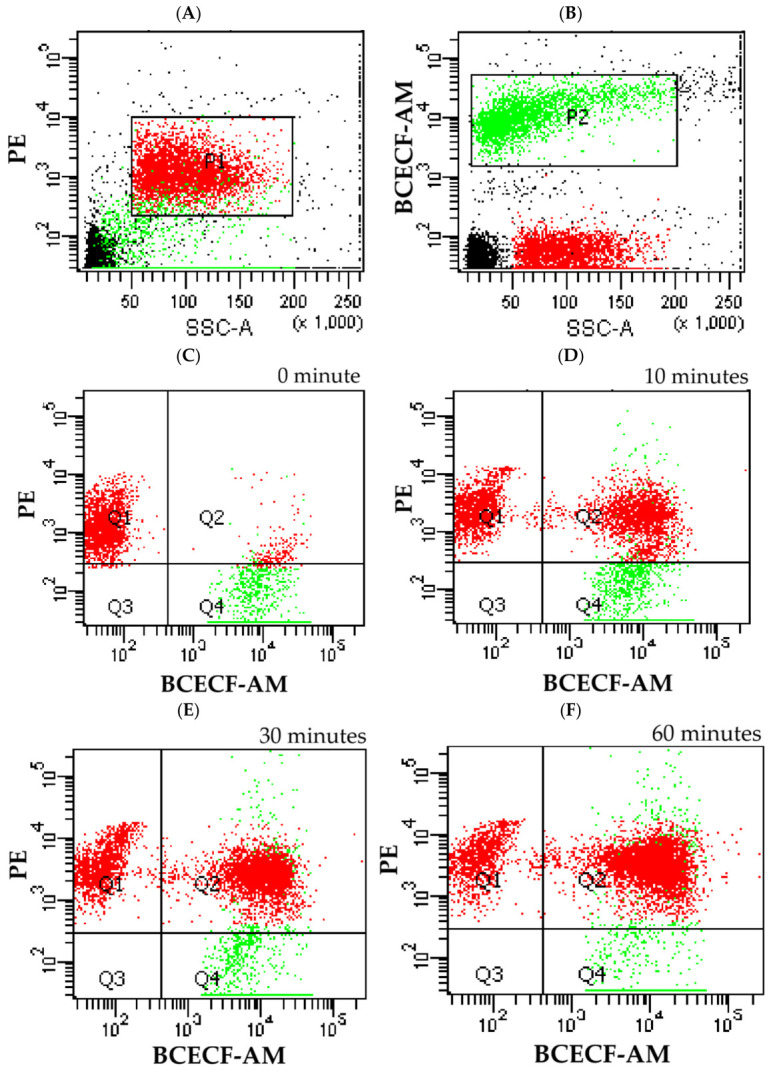
Representative flow cytometric scattergrams showing fluorescence intensity (FI) of neutrophil phagocytosis of *Candida albicans* (*C. albicans*) labeled with phycoerythrin (PE)-conjugated monoclonal antibody (**A**) and 2′,7′-bis-(2-carboxyethyl)-5-(and-6)-carboxyfluorescein acetoxymethyl ester (BCECF-AM) (**B**). Engulfed neutrophils and FI at 0, 10, 30, and 60 min are indicated in quadrant 2 (Q2) (**C**–**F**).

Figure 4 illustrates the effects of MPEE treatment on neutrophil phagocytotic activity using flow cytometric analysis. Representative scattergrams demonstrate the interaction between neutrophils and *Candida albicans*. Accordingly, phycoerythrin (PE)-conjugated monoclonal antibody (Figure 4A) and 2′,7′-bis-(2-carboxyethyl)-5-(and-6)-carboxyfluorescein acetoxymethyl ester (BCECF-AM) fluorescent dye (Figure 4B) were expressed. The PE-based detection can be indicative of both surface-bound and internalized organisms, whereas BCECF-AM can more specifically indicate internalized *Candida*, thereby confirming true phagocytosis. Engulfed neutrophils are primarily identified within quadrant 2 (Q2), which represents double-positive cells and serves as the key indicator of phagocytic activity. Across Figure 4C–F, a time-dependent increase in fluorescence intensity (FI) and Q2 cell population is observed, indicating progressive engulfment of *C. albicans* by neutrophils. This trend suggests that MPEE treatment enhanced neutrophil phagocytic function, leading to the increased uptake and processing of pathogens. Overall, the data support the conclusion that MPEE positively modulates innate immune responses by promoting efficient neutrophil-mediated phagocytosis.

**Figure 5:**
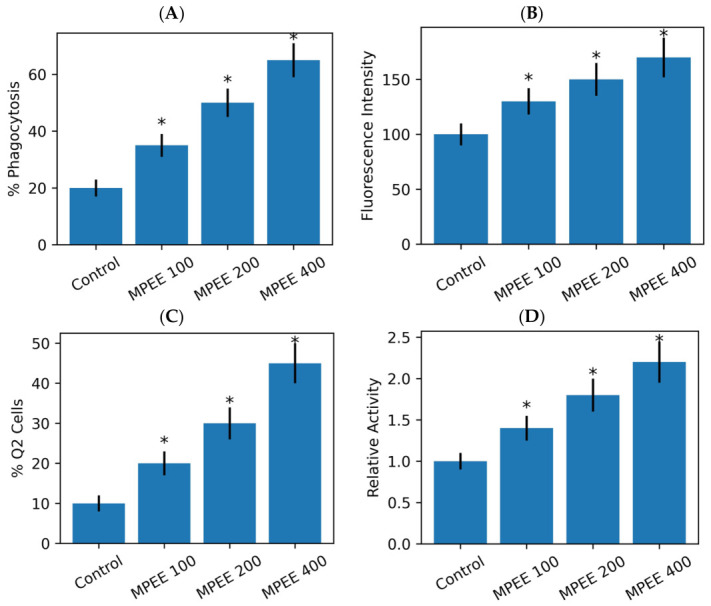
Quantitative analysis of the percentage of *C. albicans*-engulfed neutrophils treated with DI (control) and MPEE (100, 200, and 400 mg/kg) for 60 min (**A**–**D**). Data are expressed as mean ± SEM values (*n* = 12). Statistical analysis was performed using one-way ANOVA followed by Tukey’s post hoc test. Accordingly, * *p* < 0.05 when compared with the control. Abbreviation: *C. albicans*, *Candida albicans*.

Quantitative analysis revealed dose-dependent increases (*p* < 0.05) in the percentages of phagocytosing *C. albicans* by neutrophils in MPEE (100, 200, and 400 mg/kg)-treated groups when compared with DI controls (Figure 5A). This effect was accompanied by increased FI (Figure 5B,C), suggesting enhanced intracellular processing of the pathogen. These findings indicate that MPEE enhanced innate immune function (Figure 5D) by promoting neutrophil-mediated phagocytosis.

**Figure 6:**
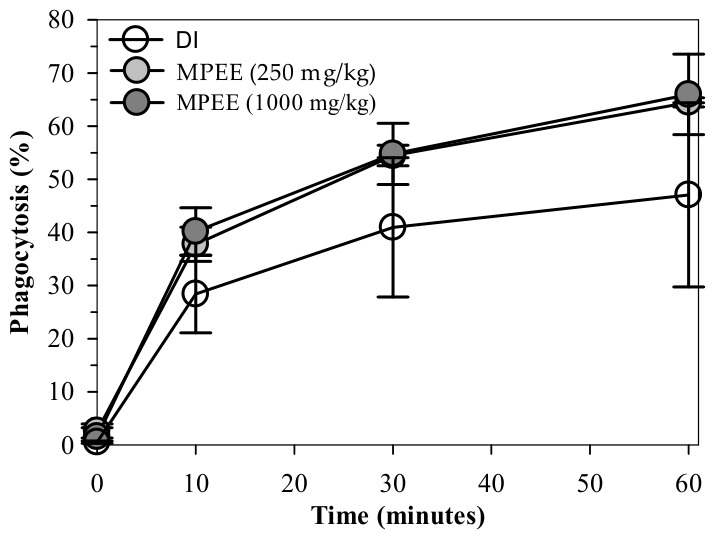
Phagocytotic activity of neutrophils from mice fed with DI or MPEE (250 and 1000 mg/kg) for 180 days. Data are presented as mean ± SD values (*n* = 12). Abbreviations: DI, deionized water; MPEE, *Mangifera indica* (Mahajanaka) pulp ethanolic extract.

The phagocytotic activity of neutrophils isolated from mice administered DI or MPEE at doses of 250 and 1000 mg/kg (*n* = 12 each) over a 180-day period are also shown in Figure 6. Interestingly, neutrophils from MPEE-treated mice increased phagocytotic activity when compared with the PBS control at equivalent concentrations, but not significantly, indicating an enhancement of innate immune function. However, a higher dose (1000 mg/kg) did not indicate any greater increase relative to the lower dose (250 mg/kg), suggesting a dose-independent effect. These findings demonstrate that prolonged MPEE administration significantly enhanced neutrophil-mediated phagocytosis.

### 2.7. Effects on Splenocyte Subpopulations

**Figure 7:**
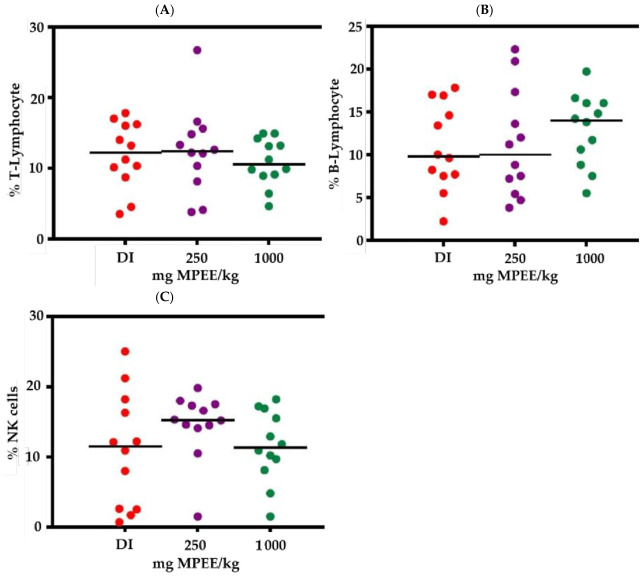
Subpopulations of splenic T-lymphocytes (**A**), B-lymphocytes (**B**), and natural killer (NK) cells (**C**) isolated from control mice treated with DI or MPEE (250 and 1000 mg/kg) for 180 days. Red colored dot represents an individual animal, and the horizontal black line represents the group mean. Data are expressed as individual values and mean ± SD values (*n* = 12). Abbreviations: DI, deionized water; MPEE, *Mangifera indica* (Mahajanaka) pulp ethanolic extract.

In our findings, we observed the distribution of splenic T-lymphocytes, B-lymphocytes, and natural killer (NK) cells in mice following the long-term administration of DI or MPEE at doses of 250 and 1000 mg/kg (*n* = 12 each) for 180 days (Figure 7). The data suggest that MPEE treatment modulated immune cell populations, with treated groups showing apparent increases in T-lymphocytes, B-lymphocytes, and natural killer (NK) cells when compared with the control group (*p* > 0.05). A greater effect was observed at the higher dose (1000 mg/kg), indicating a potential dose-related trend. However, in the absence of explicitly reported statistical significance, these differences should be interpreted with caution.

### 2.8. Effects of MPEE Treatment on Proliferation of Mitogen-Stimulated Splenocytes

Results in Table 7 reveal the stimulation index (SI) of splenocytes from mice treated with DI or MPEE (250 and 1000 mg/kg) following stimulation with Concanavalin A (ConA) at 1 and 2 μg/mL. The SI values of the DI group remain close to 1.0, indicating baseline T-cell proliferation. MPEE at 250 mg/kg showed a slight increase in SI at 1 μg/mL ConA; nonetheless, the large SD suggests considerable variability among samples. At 2 μg/mL, the SI is comparable to that of the control. In contrast, MPEE at 1000 mg/kg resulted in consistently lower SI values at both ConA concentrations, suggesting a reduction in T-cell proliferative response. These findings indicate that while low-dose MPEE may exert a modest and inconsistent stimulatory effect, higher doses may suppress T-cell activity. Overall, the results suggest a dose-dependent modulation of splenocyte proliferation, although the absence of statistical significance warrants cautious interpretation.

### 2.9. Effects on Splenocyte Cytokine Secretion upon Mitogen Stimulation

**Figure 8:**
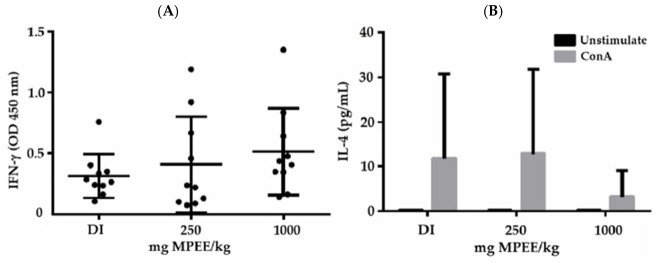
Levels of interferon-gamma (IFN-γ) (**A**) and interleukin-4 (IL-4) (**B**) secreted from the splenocytes of mice with and without stimulation at 2 μg/mL. Herein, results are presented for ConA stimulation and in mice treated with DI and MPEE (250 and 1000 mg/kg) for 180 days. Data are expressed as individual values and mean ± SD values (*n* = 9). Abbreviations: ConA, concanavalin A; DI, deionized water; MPEE, *Mangifera indica* (Mahajanaka) pulp ethanolic extract.

Figure 8 illustrates the levels of IFN-γ and IL-4 secreted by splenocytes isolated from mice treated with DI or MPEE (250 and 1000 mg/kg) for 180 days, in the presence or absence of ConA stimulation, while IFN-γ and IL-4 served as representative cytokines of Th1/Th2-mediated immune responses, respectively. As expected, ConA stimulation enhanced cytokine production when compared with unstimulated conditions. MPEE treatment modulated cytokine secretion, with alterations observed in both IFN-γ (Figure 8A) and IL-4 (Figure 8B) levels relative to the control group. Changes in IFN-γ suggest an effect on cell-mediated immunity, whereas variations in IL-4 reflect modulation of humoral immune responses. The inclusion of individual data points alongside the mean ± SD values would indicate variability among samples.

Overall, these findings suggest that long-term MPEE administration influenced T-helper cell responses and may have altered the balance between Th1 and Th2 immunity, depending upon the dose and stimulation conditions.

## 3. Discussion

The present study demonstrates that MPEE exerts multifaceted biological activities, including antihyperglycemic, hypolipidemic, antihypertensive, and immunomodulatory effects. Importantly, this study provides a novel integrative evaluation of MPEE across metabolic, cardiovascular, and immune systems within a single experimental framework. The HPLC/ESI-MS profile of MPEE demonstrates a diverse composition of phytochemicals, predominantly belonging to xanthones, flavonoids, phenolic acids, and benzophenone derivatives. The early-eluting peaks correspond to highly polar compounds, which are typically phenolic acids and glycosylated flavonoids. The detection of *m*/*z* 478 supports the presence of MGF or its derivatives, a bioactive compound widely reported in mango pulp [21,22]. The presence of ions at *m*/*z* 277 and 289 suggests flavanol compounds such as catechin and epicatechin, which are known contributors to antioxidant activity in fruits [23]. Similarly, the detection of *m*/*z* 463 and related ions indicates quercetin glycosides, which are commonly reported in mango and are associated with anti-inflammatory and promoting properties [24]. Mid-retention time peaks showing ions at *m*/*z* 597, 581, and 499 suggest the presence of complex glycosylated xanthones and galloylated derivatives. These compounds are consistent with previously reported mango phytochemicals, including MGF conjugates and related polyphenols [25]. The observation of ions in the range *m*/*z* 433–465 further indicates the presence of benzophenone glycosides such as iriflophenone derivatives, which are characteristic constituents of mango tissues [26]. In the later retention time region, the presence of ions at *m*/*z* 449 and 485 supports the occurrence of flavonoid aglycones such as quercetin and kaempferol derivatives. The detection of higher-molecular-weight ions (*m*/*z* 565–595) suggests more complex phenolic conjugates, possibly involving glycosylation or acylation processes. Overall, the phytochemical composition observed in this study is in good agreement with previously reported profiles of *M. indica* pulp. These compounds are known to contribute significantly to the antioxidant, anti-inflammatory, and nutraceutical properties of mango [22,25]. However, it is important to note that the compound identifications presented are tentative and based solely on mass spectral data obtained under positive electrospray ionization conditions. Definitive identification requires further confirmation using tandem mass spectrometry, high-resolution mass spectrometry, and comparison with authentic standards.

Regarding chemical contents, MPEE contains a high TPC of 339 ± 8.9 mg GAE/g extract, indicating a strong presence of bioactive compounds. Phenolic compounds are widely reported as major contributors to antioxidant activity due to their redox properties, allowing them to act as reducing agents, hydrogen donors, and singlet oxygen quenchers, aligning with the importance of phenolics in antioxidant evaluation and their central role in plant-based antioxidant systems. MGF (0.55 ± 0.96 mg/g extract), although present in smaller quantities, is a well-known xanthone with significant antioxidant, anti-inflammatory, and pharmacological properties. A previous study has reported that MGF exhibits strong free-radical-scavenging and metal-chelating activities, suggesting that even low concentrations can contribute meaningfully to overall antioxidant potential through synergistic interactions [1]. The L-ascorbic acid content (0.39 ± 0.13 mg/g extract) further enhances the antioxidant profile. Ascorbic acid is a primary water-soluble antioxidant that directly scavenges reactive oxygen species and regenerates oxidized antioxidants and it can significantly improve the antioxidant defense system, particularly when combined with other phytochemicals [27]. β-Carotene, recorded at 5.87 ± 0.06 mg/g extract, represents a major lipophilic antioxidant in the extract. It is known for its ability to quench singlet oxygen and protect lipid membranes from oxidative damage. Krinsky and Johnson (2005) have revealed the role of carotenoids in preventing oxidative stress-related damage, especially in lipid-rich environments [28].

Herein, the selected MPEE doses differed among experimental models because they were optimized according to the biological endpoint under investigation, experimental duration, and previously established protocols. Therefore, direct comparisons of dose–response relationships among metabolic, cardiovascular, and immunological studies should be interpreted with caution. The antioxidant activity measured using the ABTS assay (740 ± 8 mg TE/g extract) is notably high, suggesting strong radical-scavenging capacity. This result is consistent with the well-established correlation between phenolic content and antioxidant activity, as described by Re et al. (1999) [29], who demonstrated that ABTS radical-scavenging reflects the combined action of hydrophilic and lipophilic antioxidants. Overall, the strong antioxidant activity of MPEE can be attributed to the synergistic effects of its phytochemical constituents, particularly phenolics, MGF, ascorbic acid, and β-carotene. Similar synergistic interactions have been emphasized by Prior et al. (2005) [30], who noted that complex mixtures of antioxidants often exhibit greater activity than isolated compounds. The coexistence of both hydrophilic and lipophilic antioxidants in MPEE enhances its functional potential across different biological systems. Previous studies have reported on the antioxidant and biological activities of mango extracts [20], but the current findings extend this knowledge by demonstrating long-term immune–metabolic modulation, which is increasingly recognized as a key therapeutic target in chronic diseases.

In the diabetic model, MPEE exhibited a modest antihyperglycemic effect, while more pronounced improvements were observed in plasma triglycerides and total cholesterol levels, suggesting a stronger role in lipid regulation. This aligns with previous reports showing that mango-derived polyphenols, including MGF, improve lipid metabolism and insulin sensitivity by modulating oxidative stress and inflammatory pathways [1,2]. These findings support the concept that plant extracts may exert greater benefits on the metabolic complications of diabetes, such as dyslipidemia, rather than by directly lowering blood glucose levels. The modest increase in antioxidant capacity observed in this study further supports a contributory role of oxidative stress modulation, consistent with prior findings on mango phytochemicals [20]. The cardiovascular effects observed in this study also support the therapeutic potential of MPEE. The extract significantly reduced blood pressure in L-NAME-induced hypertensive rats, suggesting a role in endothelial function and vascular regulation. This outcome is consistent with those of previous studies that demonstrated that plant-derived compounds could improve nitric oxide bioavailability and reduce vascular resistance [12]. Additionally, polyphenolic compounds, such as MGF, have been shown to exert vasodilatory and antihypertensive effects through antioxidant and anti-inflammatory mechanisms [9,13,31]. The selective effect of MPEE under hypertensive conditions highlights its potential as a pathophysiology-targeted therapeutic agent, which represents an important advantage over non-specific interventions.

A major novel contribution of this study is the demonstration that MPEE enhanced innate immune function, particularly neutrophil phagocytosis. The observed increase in phagocytic activity and fluorescence intensity is indicative of improved pathogen uptake and intracellular processing. These findings are consistent with established methodologies for evaluating phagocytic function [32] and align with the outcomes of previous studies reporting that plant extracts could enhance macrophage and neutrophil activity [14]. This suggests that MPEE may strengthen first-line immune defense mechanisms, which are critical in the protection against infections. In contrast, the effects of MPEE on adaptive immunity appear more complex. Although slight increases in splenic T-, B-, and NK-cell populations were observed, these changes were not statistically significant. Similarly, splenocyte proliferation exhibited a dose-dependent biphasic response, with mild stimulation at lower doses and suppression at higher doses. This pattern is consistent with previous findings, wherein botanical extracts could exert dual immunomodulatory effects, thereby enhancing immune responses at low concentrations while suppressing excessive activation at higher doses [15,16]. Such bidirectional regulation may be beneficial in maintaining immune homeostasis and preventing overactivation. Cytokine analysis further supports this regulatory role. IFN-γ and IL-4, representing Th1 and Th2 responses, respectively, were modulated by MPEE treatment. This suggests that the extract may influence the balance between cell-mediated and humoral immunity, rather than simply enhancing one pathway. Similar immunoregulatory effects have been reported for herbal formulations that restore immune balance in aged or immunocompromised models [17]. Therefore, MPEE may function as an immune-balancing agent, thereby contributing to overall immune stability.

In terms of its benefits, this study provides several key contributions. It offers a novel multi-system evaluation of MPEE, integrating metabolic, cardiovascular, and immunological outcomes within a single study. Accordingly, the use of both functional immune assays and the relevant physiological measurements could strengthen the validity of the findings. Additionally, the long-term treatment design enhanced the translational relevance of the results. Importantly, the study identifies MPEE as a potential natural immune–metabolic modulator, which is highly relevant for managing complex chronic diseases. Despite these strengths, several limitations should be acknowledged. Some immunological findings lack statistical significance, thereby limiting the strength of our conclusions. Accordingly, high variability in certain datasets could reduce confidence in some results. Inconsistencies in dosing regimens and experimental conditions may affect comparability. Notably, this study did not identify the specific bioactive compounds responsible for the observed effects, nor did it investigate any potential underlying molecular mechanisms. Future studies should employ high-performance liquid chromatography/tandem mass spectrometry (HPLC-MS/MS) or HPLC-quadrupole time-of-flight/mass spectrometry (HPLC-QTOF/MS) and authentic standards for definitively identifying and characterizing active phytochemicals. The ABTS assay used in this study provides a broad assessment of the radical-scavenging capacity of both hydrophilic and lipophilic antioxidants of the MPEE. However, additional antioxidant assays, including 2,2-diphenyl-1-picrylhydrazyl, ferric-reducing antioxidant power and oxygen radical absorbance capacity assays, could provide complementary information regarding different antioxidant mechanisms and should be considered further. Moreover, a standard antihypertensive drug (positive control) was not included in the L-NAME-induced hypertension model, while the antihypertensive effects of MPEE were demonstrated by comparison with untreated hypertensive and normotensive control groups. Furthermore, studies should incorporate established antihypertensive agents to facilitate direct comparisons and elucidate the therapeutic potential of MPEE. The studies should also elucidate their mechanisms of action. Investigations into molecular pathways, including oxidative stress, inflammation, and endothelial function, are needed. Larger, well-powered studies should confirm the immunological findings, and clinical trials will be essential to determine translational potential. Additionally, exploring synergistic effects with existing therapies may further enhance the applicability of MPEE.

## 4. Materials and Methods

### 4.1. Chemicals and Reagents

BCECF-AM (Product number: B8806), ConA (Product number: L7647), ABTS (Product number: A1888), β-carotene (Product number: C9750), 2,6-dichlorphenolindophenol (DCPIP) (Product number: D1878), dimethyl sulfoxide (DMSO) (Product number: D2650), fetal calf serum (FCS) (Product number: F7524), GBA (Product number: G0639, >99% purity), phytohemagglutinin (PHA) (Product number: 526511, >95% purity), RPMI 1640 medium (Product number: R8758), [3-(4,5-dimethylthiazol-2-yl)-2,5-diphenyltetrazolium bromide] (MTT) (Product number: 475989), L-NAME (Product number: 483125M), phosphate-buffered saline pH 7.4 (PBS) (Product number: P4474), pentobarbital sodium (Product number: P3761), and STZ (Product number: 572201, >95% purity) were obtained from Sigma-Aldrich Chemicals Company Limited (Saint Louis, MO, USA). FITC-conjugated monoclonal anti-mouse CD3 (Product number: F-7275) was purchased from the Sigma-Aldrich Chemicals Company. Phycoerythrin-Cy7 (PE-Cy7)-conjugated monoclonal anti-mouse CD45RA (Catalogue number: 337186) was obtained from Becton-Dickinson Company, Franklin Lakes, NJ, USA and FITC-conjugated anti-mouse CD161a (Product number: FITC-65138100UG) was acquired from ThermoFisher Scientific Company Limited, Waltham, MA, USA. Enzyme-linked immunosorbent assay (ELISA) kits, for the quantification of IFN-γ (Catalogue number: E-EL-R0009) and CellaQuant™ IL-4 (Catalogue number: CQR007), were purchased from Elabscience Bionovation Inc., Houston, TX, USA).

### 4.2. Plant and Pulp Extract Preparations

MPEE was prepared according to the protocol established by Paradee and coworkers [20]. Briefly, 5 kg of fresh yellow mango fruits (*Mangifera indica*) cv. Mahajanaka (Voucher specimen number 0023368) was purchased from a local market at Doi Lo District, Chiang Mai, Thailand, in summer (10–12 April 2012). The fruit was then washed and peeled. Subsequently, the pulp of the fruit was chopped, pulverized, and extracted with 70% (*v*/*v*) ethanol at a ratio of 1:1 (*w*/*v*) at room temperature for 24 h. Afterward, the pulverized pulp was passed through a food-grade nylon sieve with a mesh size of 200 (100 μm). The extraction solution was centrifuged at 8000 rpm for 15 min. The supernatant was filtered through a solvent-resistant filter paper (Cellulose type, Whatman Grade 4, Product number WHA1004125, Maidstone, UK), while ethanol in the filtrate was removed at 50 °C using a rotatory evaporator (Drawell International Technology Limited Company, Chongqing, China). Afterward, the residual water was removed to obtain complete dryness using a freeze–dry lyophilizer. Finally, all the MPEE powder was weighed, totaling 0.325 g (6.5% yield by weight), and kept in a plastic bottle at −20 °C for further experiments.

### 4.3. Chemical Composition Analysis

#### 4.3.1. Characterization of Phenolic Compounds

Phenolic compounds were analyzed using HPLC/ESI-MS method [20,33]. The HPLC system (Agilent Technologies 1100 Series, Deutschland GmbH, Waldbronn, Germany) consisted of a quaternary pump (G1311A), an online vacuum degasser (G1322A), an autosampler (G1313A), a thermostated column compartment (G1316A) and PDA detector (G1315A). The outlet of the PDA was coupled directly to the atmospheric pressure ESI interface of the MS detector (Agilent Technologies 1100 LC/MSD SL, Palo Alto, CA, USA) through a flow splitter (1:1). The HPLC system comprised a column (LiChroCART RP-18e, 150 mm × 4.6 mm, 5 µm particle size; Purospher STAR, Merck, Darmstadt, Germany) that was regulated thermally at 40 °C. Firstly, 10 mg of MPEE powder was reconstituted in 1 mL of methanol and the sample solution was filtered through an Acrodisc membrane syringe filter (polytetrafluoroethylene type, 0.45 μm pore size, 13 mm diameter, Merck Millipore Limited Company, Burlington, MA, USA) to remove particulate matter. Then, 10 μL of the filtrate was injected into the HPLC system and eluted with mobile-phase solvent (A = acetonitrile, B = 10 mM formate buffer pH 4.0) using gradient-elution program (0–5 min: constant 100% B; 5–10 min: 0–20% A; 10–20 min: constant 20% A; 20–60 min: 20–40% A) at a flow rate of 1.0 mL/min, and detection of OD was recorded at 270 and 500 nm. Available authentic standards for GA, catechins, rutin, isoquercetin, hydroquinine, eriodictyol, apigenin and kaempherol were included for the purposes of identifying all phenolic compounds. MS analysis was performed in positive ESI mode, and mass spectra were acquired within the mass-to-charge ratios (*m*/*z*) ranging from 100 to 700. For the single quadrupole MS system, the ESI energy was set at 70 eV, while the temperatures of the ion source and the interface were set at 150 °C and 230 °C, respectively. Nitrogen was employed as the nebulizing, drying and collision gas. The capillary temperature was set at 320 °C, the nebulizer pressure was set to 60 pounds/inch^2^ and the drying gas flow rate was set to 13 L/min. Capillary voltages were set to 3500 V (positive) and 150 V (negative). The oven temperature was programmed as follows: 80 °C (held for 3 min), ramped to 110 °C at 10 °C/min (held for 5 min), increased to 190 °C (held for 3 min), ramped to 220 °C at 10 °C/min (held for 4 min), and increased to 280 °C at 15 °C/min (held for 13 min). Accurate mass measurement was performed using the auto mass calibration method with an external mass calibration solution (ESI-L Low-Concentration Tuning Mix; Agilent calibration solution B). Herein, the low limits of detection, the limits of quantitation and the relevant recovery values were found to be 0.5 mg/kg, 1.20 mg/kg and 70–110%, respectively. The chromatographic and MS analysis and prediction of the chemical formula, including the exact mass calculation, were performed by Mass Hunter software version B.04.00 build 4.0.479.0 (Agilent Technology). In the assay process, the MPEE was constituted in 1.0 mL of A: B mixture (equal volume) and injected into the HPLC-MS system for analysis of polar phenolic compounds. The detected peaks were evaluated according to retention time and *m*/*z* values. Tentative compound identification was performed by comparing observed mass spectral signals with the available authentic standards and reported phenolic compounds commonly found in mango pulp (e.g., phenolic acids, xanthones, flavan-3-ols and flavonol glycosides).

#### 4.3.2. Determination of TPC

TPC was measured in three separate experiments using Folin–Ciocalteu reagent in alkaline conditions [34]. MPEE (10 mg/mL) or standard GA (0.1 mL) was mixed with Folin–Ciocalteu reagent (0.1 mL) and 7% (*w*/*v*) sodium carbonate (1.0 mL) and incubated at room temperature for 30 min and measured optical density (OD) of the product at 630 nm against reagent blank using the UV-VIS spectrophotometer. TPC was determined from a standard curve of GA (8.5–170 μg/mL) and reported as GAE.

#### 4.3.3. Determination of L-Ascorbic Acid Content

L-Ascorbic acid content in MPEE was determined in triplicate using the DCPIP titration method [35]. 50 mL of MPEE solution (10 mg/mL) was mixed with 3% phosphoric acid in DI and then titrated with 0.1% (*w*/*v*) DCPIP solution until a pink color appeared. The volume of DCPIP was recorded. L-ascorbic acid concentration was calculated from the standard.

#### 4.3.4. Determination of β-Carotene Content

β-Carotene content was determined twice using the HPLC-DAD method as previously established by Dzakovich et al. [36]. In brief, MPEE (100 mg) was saponified with 10 mL of 10% (*w*/*v*) potassium hydroxide in ethanol at 85 °C for 30 min, followed by extraction with n-hexane (2 × 5 mL). The extract was concentrated using rotary evaporators. The sample was reconstituted with methanol and injected into the Agilent HPLC-DAD system. Fractionation occurred on a column (C18 type, 4.6 mm × 250 mm, 5 μm particle size), with elution by a mobile-phase solvent composed of acetonitrile-methanol-ethyl acetate (88:10:2) at a flow rate of 1 mL/min. Eluents were detected at a wavelength of 436 nm with a DAD. The results were expressed as mg β-carotene/100 g fresh weight.

#### 4.3.5. Quantification of MGF

MGF (10 mg/mL) was quantified twice using the HPLC-DAD system (Agilent Technologies, Santa Clara, CA, USA) with the condition included a column (LiChroCART RP-18e, Product number: 1.50358.0001, 250 mm × 4.6 mm, 5 µm particle size; Purospher STAR, Merck, Darmstadt, Germany), the mobile-phase solvent composing methanol:3% acetic acid (33:67, *v*/*v*), a flow rate of 0.5 mL/min, and photodiode array detection at 254 nm [37]. MGF concentration was determined from a standard curve of MGF.

### 4.4. Dose Selection and Experimental Design

Because the present work integrated data from several experimental models that were originally designed to investigate different biological endpoints, identical dose ranges were not used across all experiments.

### 4.5. Assay of Antioxidant Activity

Determination of antioxidant activity is based on the ability of a compound or standard Trolox (water-soluble vitamin E analogue) to decolorize ABTS^•+^ to a colorless ABTS product [38]. Briefly, mango extract (10 mg/mL) or standard Trolox (0.1 mL) was incubated with ABTS^•+^ solution (2 mL) at room temperature for 30 min and measured OD values at 734 nm against reagent blank using a double-beam UV/VIS spectrophotometer (Model 1900, Shimadzu Corporation, Kyoto, Japan). Antioxidant activity was determined in three separate experiments and calculated from a standard curve of Trolox (25–500 μg/mL) and reported as mg TE/g extract.

### 4.6. Experimental Animals and Ethical Approval

Male Sprague-Dawley rats weighing 180–200 g were obtained from the National Laboratory Animal Center, Mahidol University, Salaya, Nakhon Pathom Province, Thailand. The animals were housed in a temperature-controlled environment (22 ± 3 °C) under a 12-h light/12-h dark cycle. Standard laboratory chow was purchased from Perfect Companion Group Company Limited, Samutprakarn, Thailand. Food and water were provided *ad libitum*. All experimental procedures were conducted in accordance with the Code of Ethics for the Use of Experimental Animals of the National Research Council of Thailand (1999). The study protocol was approved by the Animal Ethics Committee, Faculty of Medicine, Chiang Mai University (Project Code 21/2554).

### 4.7. Study of Anti-Diabetic Activity in Rats

#### 4.7.1. Blood Collection and Preparation

Prior to weekly administration, animals were fasted for 8–12 h. Approximately 0.5 mL of blood from the tail vein was collected into heparinized tubes. The plasma was separated by centrifugation at 3000 rpm, 4 °C for 15 min for the determination of FBG and plasma lipid profiles. At the end of the experimental period, rats were euthanized with an IP injection of pentobarbital sodium (75 mg/kg). Blood samples were collected via cardiac puncture from the left ventricle into heparinized tubes, and the plasma was separated for biochemical analysis.

#### 4.7.2. Induction of Diabetes in Rats and Treatment

Diabetic mellitus (DM) was induced in N rats using STZ, which had been previously dissolved in 10 mM citrate buffer (pH 4.0) and administered as a single subcutaneous (sc) injection of STZ at a single dose of 65 mg/kg BW. After one week, the rats were fasted for 8–12 h and approximately 0.1 mL of blood was collected from the tail veins for the measurement of FBG levels. Rats with FBG levels between 200–220 mg/dL were considered DM and selected for the study. Accordingly, the animals were divided into seven groups and orally (po) administered with DI or MPEE as follows. Group 1: N rats (*n* = 11) received DI (0.5 mL/day); Group 2: N rats (*n* = 6) received MPEE (30 mg/kg/day); Group 3: N rats (*n* = 6) received MPEE (300 mg/kg); Group 4: DM rats (*n* = 6) received DI; Group 5: DM rats (*n* = 6) received GBA (3 mg/kg/day) for 6 weeks; Group 6: DM rats (*n* = 11) received MPEE (30 mg/kg/day); and Group 7: DM rats (*n* = 11) received MPEE (300 mg/kg/day) for 6 weeks.

#### 4.7.3. Biochemical Measurements

##### Plasma Glucose

Plasma glucose concentrations were determined with the use of a glucose oxidase/peroxidase reagent kit following the manufacturer’s instructions. In the assay, 10 µL of the plasma was mixed with 3.0 mL of glucose enzymatic reagent. The mixture was then incubated at room temperature for 15 min, and optical density (OD) was measured at 495 nm. Glucose concentrations were calculated using a standard calibration curve (0–400 mg/dL).

##### Plasma Triglyceride

Plasma TG was determined with the use of a lipase/glycerol oxidase/peroxidase reagent kit following the manufacturer’s instructions. In the assay, 100 µL of the plasma was mixed with 3.0 mL of the triglyceride enzymatic reagent. After incubation at room temperature for 15 min, OD was measured at 495 nm. TG concentrations were then calculated using a standard curve (0–400 mg/dL).

##### Plasma Total Cholesterol

Plasma TC was determined with the use of a cholesterol esterase/cholesterol oxidase/peroxidase reagent kit following the manufacturer’s instructions. In the assay, 100 µL of the plasma was mixed with 3.0 mL of the cholesterol enzymatic reagent. The mixture was then incubated at room temperature for 15 min, and the OD was measured at 495 nm. TC concentration was determined using a standard calibration curve (0–400 mg/dL).

### 4.8. Study of Effects of MPEE on Blood Pressure and Heart Rate in STZ-Induced Rats

#### 4.8.1. Normal Blood Pressure Model

Normal rats were randomly divided into four groups (*n* = 5 each) and treated as follows. Group 1 orally received DI, while Groups 2, 3, and 4 received MPEE at 100, 200, and 400 mg/kg, respectively. The rats were anesthetized with pentobarbital sodium (35 mg/kg, ip). An incision was made in the neck for endotracheal intubation. A polyethylene cannula was inserted into the jugular vein for the administration of the test substances, while another polyethylene cannula was inserted into the femoral artery for recording BP and HR values. Measurements were monitored via a ventilator-assisted setup [12].

#### 4.8.2. L-NAME-Induced Hypertension Model

The rats were also divided into four groups (*n* = 5 each): Group 1 orally received DI, while Groups 2, 3, and 4 received MPEE at 100, 200, and 400 mg/kg, respectively. The rats were anesthetized with pentobarbital sodium (35 mg/kg, ip). An incision was made in the neck for endotracheal intubation. A polyethylene cannula was inserted into the jugular vein for the administration of the test substances, and another was inserted into the femoral artery to record BP and HR values. Hypertension was induced in the anesthetized rats by iv injection of L-NAME at a dose of 3 mg/kg through the jugular vein. Approximately 15 min later, the test substance was administered intravenously [12].

### 4.9. Study of Effects of MPEE on Immune Cells

Splenocytes (immune cells) were isolated from the spleen and peripheral blood of mice (*n* = 12 each) that had been orally administered with MPEE (100, 200 and 400 mg/kg) for 180 days. The effects on immune function were evaluated as follows.

#### 4.9.1. Splenocyte Subpopulation Analysis

Lymphocyte subpopulations were determined by staining splenocytes with FITC-conjugated anti-mouse CD3 (T lymphocytes), PE-Cy7-conjugated anti-mouse CD45RA (B lymphocytes), and FITC-conjugated anti-mouse CD161a (natural killer cells). The stained cells were analyzed using a BD FACSLyric™ flow cytometer (Becton Dickinson Company, Franklin Lakes, NJ, USA), and data were processed using BD FACSDiva software version 9.0 run on the Microsoft™ Windows™ 10 64-bit operating system [18].

#### 4.9.2. Splenocyte Proliferation Assay

Splenocytes were cultured in RPMI1640 medium (ThermoFisher Scientific Company Limited, Waltham, MA, USA) supplemented with 10% (*v*/*v*) FCS in the presence of varying concentrations of PHA or ConA [15,16]. The cultures were incubated at 37 °C for 68 h. After incubation, the culture medium was removed, and the cells were resuspended in 100 µL of PBS. Subsequently, 10 µL of 12 mM MTT solution was added to each well, and the solution was then incubated at 37 °C for 4 h. Subsequently, the plates were centrifuged at 2000 rpm for 10 min, the supernatant was discarded, and 50 µL of DMSO was added to dissolve the formazan crystals. After a further incubation period at 37 °C for 10 min, the OD was measured at 540 nm. The SI value was then calculated using Equation (1):

SI = [OD_mitogen_ − OD_DMSO_]/[OD_mitogen_^−^ − OD_DMSO_] (1)

where OD_mitogen_^+^ = OD from mitogen-stimulated sample, OD_DMSO_ = OD from DMSO-treated sample, and OD_mitogen_^−^ = OD from sample without mitogen stimulation.

#### 4.9.3. Cytokine Quantification

Splenocytes were cultured in 2 mL of R10 medium containing ConA (1 µg/mL) and incubated at 37 °C for 24 h. The culture supernatants were collected for determination of IFN-γ and IL-4 using the ELISA kits according to the manufacturer’s instructions [17].

#### 4.9.4. Phagocytic Activity Assay

Whole blood (500 µL) was diluted with PBS to obtain a leukocyte suspension (2.5 × 10^6^ cells/mL). Granulocytes were labeled with anti-granulocyte-phycoerythrin (RP-1-PE), followed by centrifugation and washing with PBS. The labeled cells were then mixed with *C. albicans* yeast labeled with BCECF-AM at a 1:1 ratio of leukocytes to yeast cells. The mixture was incubated at 37 °C for 0–60 min. At 0, 10, 30, and 60 min, the reaction was terminated by adding 0.1% (*v*/*v*) paraformaldehyde buffer at a pH of 7.0. Phagocytic activity was subsequently analyzed using flow cytometry [32].

### 4.10. Statistical Analysis

Data are expressed as mean ± SD or SEM, as indicated. Statistical analysis was performed using SPSS Inc. Software version 21 (IBM Corporation, Chicago, IL, USA). Graphs were made by using SigmaPlot Program version 15 (Informer Technologies Inc., Grafiti LLC, PaloAlto, CA, USA). Differences among groups were evaluated by one-way or two-way analysis of variance (ANOVA), followed by Tukey’s post hoc test. Student’s *t*-test was used for comparisons between two groups, where appropriate. A *p*-value < 0.05 was considered statistically significant.

## 5. Conclusions

The study provides novel and comprehensive evidence that MPEE possesses diverse phytochemical constituents and exhibits multi-target biological activities. The extract demonstrated a rich composition of phenolic compounds, including xanthones, flavonoids, and phenolic acids, together with measurable levels of mangiferin, L-ascorbic acid, and β-carotene, contributing to strong antioxidant capacity. Importantly, the extract exerts multi-target biological effects, particularly by improving lipid metabolism, reducing blood pressure, and enhancing innate immune function. While its antihyperglycemic and adaptive immune effects are more modest, MPEE appears to act as an immunometabolic regulator, supporting both metabolic health and immune balance. These findings highlight its potential as a natural therapeutic or functional supplement, although further mechanistic and clinical studies would be required to confirm its efficacy and safety.

## Figures and Tables

**Table 1:** Tentative identification of phytochemical compounds in the MPEE by HPLC/ESI-MS illustrating the separation and tentative identification of phenolic compounds based on retention time (T_R_) and *m*/*z* values in positive ion mode.

Peak	T_R_ (min)	Major *m*/*z* (Approximately)	Proposed Ions (Positive Mode)	Compound Class	Tentative Compound
1	5.326	295, 327, 346, 358, 390, 478	[M+H]^+^, [M+Na]^+^	Xanthones/Phenolics	Mangiferin derivative, phenolic glycosides
2	7.702	277, 289, 313	[M+H]^+^	Flavonol	Catechin/epicatechin-like
3	11.500	265 (base), 283	[M+H]^+^	Phenolic acid	Gallic acid derivative
4	13.501	265, 463	M+H]^+^	Flavonoid	Quercetin glycoside
5	15.002	283, 298, 348, 597	[M+H]^+^	Flavonoid glycoside	Diglycosylated flavonoid
6	21.367	597 (base), 743	[M+H]^+^	Xanthone	Mangiferin gallate/complex xanthone glycoside
7	24.134	597, 571	[M+H]^+^	Phenolics	Polyphenolic glycoside
8	25.857	460, 465	[M+H]^+^	Benzophenone	Benzophenone glycoside
9	26.938	499, 468	[M+Na]^+^	Xanthones	Mangiferin derivative
10	28.922	581 (base)	[M+Na]^+^	Glycoside	Diglycoside (flavonoid/xanthone)
11	30.760	581, 727	[M+Na]^+^	Polyphenol	Phenolic glycosides
12	34.511	263, 285	Fragment	Phenolics	Phenolic fragments
13	37.920	565	[M+H]^+^	Phenolics	Polyphenol derivative
14	42.732	595 (base)	[M+Na]^+^	Phenolic	Galloylated glycoside
15	43.672	433 (base)	[M+H]^+^	Benzophenone	Benzophenone derivative
16	46.208	449 (base), 327	[M+H]^+^	Flavonoid	Quercetin glycoside
17	47.837	283, 298, 485	[M+H]^+^	Flavonoid	Kaempferol/quercetin derivative
18	56.901	300, 485	[M+H]^+^	Flavonoid	Late-eluting flavonoid

Abbreviations: MPEE, *Mangifera indica* (Mahajanaka) pulp ethanolic extract; HPLC/ESI-MS, high-performance liquid chromatography/electrospray ionization–mass spectrometry.

**Table 2:** Total phenolic content (TPC), mangiferin (MGF), β-carotene, L-ascorbic acid content, and antioxidant activity of MPEE. Data are expressed as mean ± standard deviation (SD).

Chemical Composition	MPEE
TPC (mg GAE/g extract)	339 ± 8.9
MGF (mg/g extract)	0.55 ± 0.96
L-Ascorbic acid (mg/g extract)	0.39 ± 0.13
β-Carotene (mg/g extract)	5.87 ± 0.06
Antioxidant activity (mg TE/g extract)	740 ± 8

Abbreviations: GAE, gallic acid equivalents; MPEE, *Mangifera indica* (Mahajanaka) pulp ethanolic extract; MGF, mangiferin; SD, standard deviation; TE, Trolox equivalents; TPC, total phenolic content.

**Table 3:** Effect of glibenclamide (GBA) and MPEE on levels of fasting blood glucose (FBG) of normal (N) and diabetic mellitus (DM) rats over time. Data are expressed as mean ± standard errors of the mean (SEM). Statistical analysis was performed using one-way analysis of variance (ANOVA) followed by Tukey’s post hoc test. Accordingly, *** *p* < 0.001 when compared with deionized water (DI) group; ^#^ *p* < 0.05, ^##^ *p* < 0.01 when compared with week 0.

	FBG (mg/dL)						
Time	N Rats (*n* = 6 Each)			DM Rats (*n* = 6 Each)			
(Week)	DI	MPEE (30 mg/kg)	MPEE (300 mg/kg)	DI	GBA (3 mg/kg)	MPEE (30 mg/kg)	MPEE (300 mg/kg)
0	155 ± 22	134 ± 17	146 ± 32	436 ± 62	420 ± 57	415 ± 96	406 ± 67
2	148 ± 49	116 ± 16	144 ± 29	476 ± 75	435 ± 74	430 ± 121	445 ± 146
4	129 ± 34	121 ± 26	115 ± 27	451 ± 70	327 ± 48 ^#^	442 ± 76	460 ± 83
6	124 ± 28	118 ± 19	101 ± 13	469 ± 19	270 ± 19 ***^,##^	463 ± 81	437 ± 12

Abbreviation: MPEE, *Mangifera indica* (Mahajanaka) pulp ethanolic extract.

**Table 4:** Effect of GBA and MPEE on levels of plasma triglyceride (TG) of N and DM rats over time. Data are expressed as mean ± SEM. Statistical analysis was performed using one-way ANOVA followed by Tukey’s post hoc test. Accordingly, ** *p* < 0.01 and *** *p* < 0.001 when compared with DI group; ^#^ *p* < 0.05, ^##^ *p* < 0.01 when compared with week 0.

	TG (mg/dL)						
Time	N Rats (*n* = 6 Each)			DM Rats (*n* = 6 Each)			
(Week)	DI	MPEE (30 mg/kg)	MPEE (300 mg/kg)	DI	GBA (3 mg/kg)	MPEE (30 mg/kg)	MPEE (300 mg/kg)
0	46 ± 12	45 ± 19	35 ± 7	112 ± 21	104 ± 19	111 ± 30	120 ± 39
2	45 ± 19	31 ± 10	26 ± 8	100 ± 22	82 ± 10 ^#^	73 ± 10 ^#^	73 ± 21
4	55 ± 22	35 ± 12	31 ± 9	97 ± 15	70 ± 18 ^#^	70 ± 14 ^#^	74 ± 26
6	57 ± 24	19 ± 6 ***^,##^	29 ± 11 ***	109 ± 6	67 ± 31 **^,#^	61 ± 31 ***^,##^	69 ± 52 **

Abbreviations: DI, deionized water; DM, diabetic mellitus; GBA, glibenclamide; MPEE, *Mangifera indica* (Mahajanaka) pulp ethanolic extract; N, normal.

**Table 5:** Effect of GBA and MPEE on levels of plasma total cholesterol (TC) of N and DM rats over time. Data are expressed as mean ± SEM. Statistical analysis was performed using one-way ANOVA followed by Tukey’s post hoc test. Accordingly, * *p* < 0.05, ** *p* < 0.01 when compared with diabetic control.

	TC (mg/dL)						
Time	N Rats (*n* = 6 Each)			DM Rats (*n* = 6 Each)			
(Week)	DI	MPEE (30 mg/kg)	MPEE (300 mg/kg)	DI	GBA (3 mg/kg)	MPEE (30 mg/kg)	MPEE (300 mg/kg)
0	68 ± 14	62 ± 24	76 ± 10	127 ± 20	138 ± 33	133 ± 25	129 ± 73
2	58 ± 18	64 ± 7	63 ± 9	116 ± 36	88 ± 30	101 ± 39	96 ± 44
4	52 ± 14	64 ± 9	66 ± 11	110 ± 41	90 ± 45	97 ± 22	94 ± 28
6	65 ± 15	65 ± 14	62 ± 7	117 ± 50	66 ± 13 *	73 ± 20	64 ± 17 **

Abbreviations: DI, deionized water; DM, diabetic mellitus; GBA, glibenclamide; MPEE, *Mangifera indica* (Mahajanaka) pulp ethanolic extract; N, normal.

**Table 6:** Antioxidant activity levels in plasma of total antioxidant capacity (TAC) of N and streptozotocin (STZ)-induced DM rats treated with MPEE (30 and 300 mg/kg) for 2, 4, and 6 weeks. Data are expressed as mean ± SD.

	TAC (mg TE/dL)						
Time	N Rats (*n* = 6 Each)			DM Rats (*n* = 6 Each)			
(Week)	DI	MPEE (30 mg/kg)	MPEE (300 mg/kg)	DI	GBA (3 mg/kg)	MPEE (30 mg/kg)	MPEE (300 mg/kg)
0	53.5 ± 6.8	55.7 ± 2.0	55.8 ± 8.6	55.1 ± 3.3	55.4 ± 9.8	54.7 ± 1.0	51.4 ± 4.4
2	60.1 ± 4.8	55.2 ± 6.8	55.6 ± 9.8	51.7 ± 4.5	58.5 ± 4.2	59.4 ± 3.3	61.6 ± 3.5
4	54.1 ± 4.6	52.1 ± 4.1	54.2 ± 4.1	57.3 ± 6.6	58.2 ± 2.8	53.4 ± 5.6	52.9 ± 4.2
6	67.2 ± 3.5	58.1 ± 2.4	60.8 ± 6.4	55.3 ± 6.9	58.8 ± 11.0	53.7 ± 7.7	58.9 ± 6.4

Abbreviations: DI, deionized water; DM, diabetic mellitus; GBA, glibenclamide; MPEE, *Mangifera indica* (Mahajanaka) pulp ethanolic extract; N, normal; TE, Trolox equivalents.

**Table 7:** Stimulation index (SI) of splenocytes from mice previously stimulated with concanavalin A (ConA) (1 and 2 μg/mL) and treated with DI and MPEE (250 and 1000 mg/kg). Data are presented in mean ± SD values (*n* = 9 each).

Treatment	SI Values	
	ConA (1 μg/mL)	ConA (2 μg/mL)
DI	0.99 ± 0.37	1.05 ± 0.38
MPEE (250 mg/kg)	1.29 ± 1.42	1.04 ± 0.85
MPEE (1000 mg/kg)	0.89 ± 0.35	0.90 ± 0.32

Abbreviations: DI, deionized water; MPEE, *Mangifera indica* (Mahajanaka) pulp ethanolic extract.

## Data Availability

The original contributions presented in this study are included in the article. Further inquiries can be directed to the corresponding authors.

## Abbreviations

The following abbreviations have been used in this manuscript:

ABTS	2,2-Azino-di-[3-ethylbenzthiazolinsulfonate
ANOVA	Analysis of variance
BCECF-AM	2′,7′-Bis-(2-carboxyethyl)-5-(and-6)-carboxyfluorescein, acetoxymethyl ester
BP	Blood pressure
BW	Body weight
ConA	Concanavalin A
DAD	Diode array detection
DI	Deionized water
DM	Diabetic mellitus
DMSO	Dimethyl sulfoxide
ELISA	Enzyme-linked immunosorbent assay
FBG	Fasting blood glucose
FCS	Fetal calf serum
FITC	Fluorescein isothiocyanate
GBA	Glibenclamide
HR	Heart rate
HSD	Honestly significant difference
IFN-γ	Interferon-gamma
IL-4	Interleukin 4
ip	Intraperitoneal
iv	Intravenous
MGF	Mangiferin
MPEE	*Mangifera indica* (Mahajanaka) pulp ethanolic extract
MTT	[3-(4,5-Dimethylthiazol-2-yl)-2,5-diphenyltetrazolium bromide]
N	Normal
L-NAME	N(G)-Nitro-L-arginine methyl ester] hydrochloride
NK	Neutral killer
OD	Optical density
PBS	Phosphate-buffered saline
PE	Phycoerythrin
PHA	Phytohemagglutinin
Q	Quadrant
RP-1-PE	Anti-granulocyte-phycoerythrin
sc	Subcutaneous
SD	Standard deviation
SEM	Standard errors of the mean
T_R_	Retention time
TE	Trolox equivalent
TPC	Total phenolic content
